# Canopy Interception for a Tallgrass Prairie under Juniper Encroachment

**DOI:** 10.1371/journal.pone.0141422

**Published:** 2015-11-06

**Authors:** Chris B. Zou, Giulia L. Caterina, Rodney E. Will, Elaine Stebler, Donald Turton

**Affiliations:** 1 Department of Natural Resource Ecology & Management, Oklahoma State University, Stillwater, OK 74078, United States of America; 2 Universidade Estadual Paulista, Botucatu, SP, Brasil; Oregon State University, UNITED STATES

## Abstract

Rainfall partitioning and redistribution by canopies are important ecohydrological processes underlying ecosystem dynamics. We quantified and contrasted spatial and temporal variations of rainfall redistribution for a juniper (*Juniperus virginiana*, redcedar) woodland and a tallgrass prairie in the south-central Great Plains, USA. Our results showed that redcedar trees had high canopy storage capacity (*S*) ranging from 2.14 mm for open stands to 3.44 mm for closed stands. The canopy funneling ratios (*F*) of redcedar trees varied substantially among stand type and tree size. The open stands and smaller trees usually had higher *F* values and were more efficient in partitioning rainfall into stemflow. Larger trees were more effective in partitioning rainfall into throughfall and no significant changes in the total interception ratios among canopy types and tree size were found. The *S* values were highly variable for tallgrass prairie, ranging from 0.27 mm at early growing season to 3.86 mm at senescence. As a result, the rainfall interception by tallgrass prairie was characterized by high temporal instability. On an annual basis, our results showed no significant difference in total rainfall loss to canopy interception between redcedar trees and tallgrass prairie. Increasing structural complexity associated with redcedar encroachment into tallgrass prairie changes the rainfall redistribution and partitioning pattern at both the temporal and spatial scales, but does not change the overall canopy interception ratios compared with unburned and ungrazed tallgrass prairie. Our findings support the idea of convergence in interception ratio for different canopy structures under the same precipitation regime. The temporal change in rainfall interception loss from redcedar encroachment is important to understand how juniper encroachment will interact with changing rainfall regime and potentially alter regional streamflow under climate change.

## Introduction

The encroachment of woody plants into grasslands is a worldwide phenomenon [1–3]. In North America, the grasslands of the south-central Great Plains are among the most affected, with some regions such as the Edwards plateau in Texas having completely converted into juniper woodland [1–2]. In Oklahoma and Kansas, woody plant encroachment is less advanced but is occurring at an accelerated rate and represents a serious threat to the remaining grasslands [4–7]. Redcedar (*Juniperus virginiana* L.) is the most common encroaching woody species in the south-central Great Plains in the United States [5, 8]. Its encroachment usually begins as well-spaced, cone-shaped small trees with compact canopies and large live branches low to ground. In some locations, redcedar encroachment initiates as a dense patch containing relatively thin trees and live branches mainly in the upper portion of the tree. In the south-central Great Plains, both open stands and dense stands will transition into redcedar woodlands in 40 to 50 years through coalescing of the open stands or self-thinning of the dense stands [9]. This woodland development produces a novel ecosystem with poorly understood ecological and hydrological consequences.

In arid rangeland, over 30% of annual rainfall can be lost to interception by vegetation canopies [10–11], similar to or even higher than that of many forests [12–13]. Experimental watershed studies in the south-central Great Plains showed that rangeland transformation from grassland to redcedar woodland resulted in a substantial reduction of surface runoff [14]. This reduction was attributed in part to high canopy interception by redcedar compared with the prairie grasses.

A few studies have quantified canopy interception by juniper encroaching into rangelands [15–16]. These studies concluded that a relatively high proportion of rainfall is lost to juniper canopy interception in the south-central Great Plains, with the interception ratios ranging from 35 to 52% of total rainfall on an annual basis. However, these studies did not specifically address the variability of rainfall interception loss from trees with different canopy structures, leaving large uncertainty in extrapolation from tree level measurements to the stand or landscape scale. Canopy structure metrics for trees associated with growth form (*e*.*g*., open stands vs. closed stands vs. dense stands) may affect canopy storage capacity (*S*) and canopy funneling ratio (*F*) and therefore the partition of net rainfall into throughfall and stemflow. The stemflow funneling ratio of shrub species were reported to be among the highest for woody plants [17]. It is not known whether open redcedar stands are able to effectively funnel rainfall into concentrated stemflow.

Components of canopy interception loss include evaporation from canopy storage once the event has ceased, *C*_*e*_ and evaporation from the vegetation canopy during a rainfall event, *E*_*t*_ [12,18]. *C*_*e*_ is more closely related to the canopy characteristics and may be numerically equal to *S* when rainfall is sufficiently large to satisfy the canopy storage capacity [19]. *E*_*t*_ is determined primarily by meteorological conditions such as rainfall regime and atmospheric demand during the rainfall event. We have very limited information regarding how vegetation canopy characteristics and meteorological conditions interact to influence interception loss [19–20].

There is a large uncertainty for rainfall interception loss from tallgrass prairie at various growth stages and under different management practices (e.g., burning or grazing). Previous studies reported a wide range of interception values for tallgrass prairie, ranging from 25% to 60% of bulk rainfall [21–22]. Canopy development phases for grass (early growing season vs. full canopy development vs. senescence) likely change *S* value of grass canopy.

To construct the hydrological budget and manage water resources for an ecosystem in transition, it is important to know the differences of canopy interception for the alternative vegetation covers. Contrasting rainfall loss to canopy interception to event rainfall and for a given period of time provides important insight for interpreting soil water content and streamflow responses. Increasing evidence suggests that stands with widely differing canopy characteristics may have similar interception values under the same precipitation regime [23]. However, it is not known whether this applies to differing stand structures that develop during juniper encroachment.

Our main objective was to relate rainfall amount, canopy form of redcedar trees and growing phase of tallgrass prairie to rainfall interception and redistribution. We ask the following questions: (1) How do the canopy traits of redcedar and the development phase of tallgrasses affect storage capacity (*S*) and stemflow funneling ratio (*F*)? (2) How do vegetation canopy characteristics and meteorological conditions interact to influence interception loss? (3) Does the canopy interception ratio of the tallgrass prairie vegetation change with growing stage? (4) Will rainfall loss to canopy interception increase, decrease, or remain unchanged after redcedar encroachment into tallgrass prairie? Answers to these questions are needed for extrapolating tree-level measurements to the landscape level and to improve hydrological model parameterization for modeling the impact of woody plant encroachment in south-central Great Plains [24].

## Methods

### Study site

**Fig 1:**
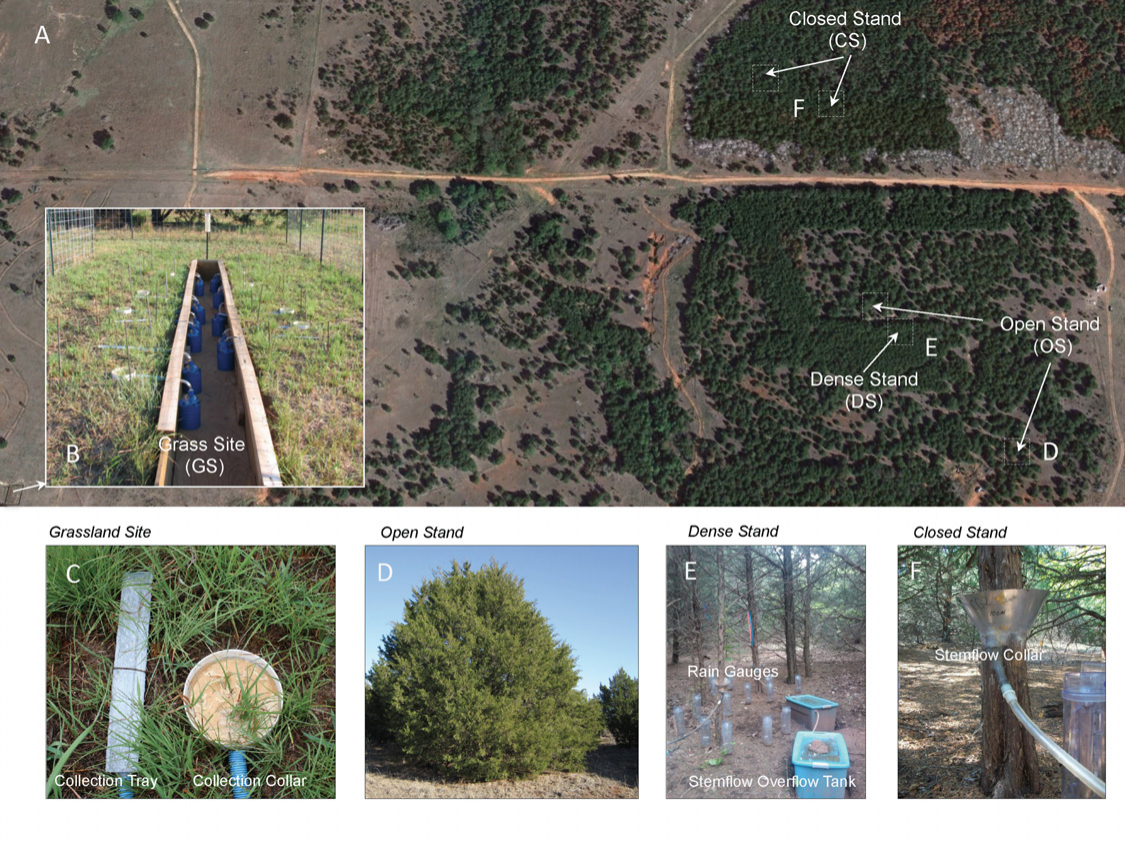
Experimental plots located in the south-central Great Plains USA at the Oklahoma State University Research Range (A) showing grassland site (B), a close-up of a grass tray interceptor (C), open stand site (D), dense stand site showing rain gauges and stemflow collection tanks (E), and closed stand site showing stemflow collector collar (F).

The study site is located 15 km southwest of Stillwater in Payne County, Oklahoma (36°04’N, 97°11’W) and managed by Oklahoma State University. Payne County’s long-term (1971–2000) average annual precipitation is 948 mm, the average annual temperature is 15.5°C, and the mean humidity is 69% [25]. Most of this area is considered tallgrass prairie with big bluestem (*Andropogon gerardii* Vitman), little bluestem [*Schizachyrium scoparium* (Michx.) Nash], indiangrass [*Sorghastrum nutans* (L.) Nash], and switchgrass (*Panicum virgatum* L.) being the dominant grass species. Historic aerial photos show that redcedars began encroaching in the late 1970s. Fire and grazing interactions through patch-burning have been studied in parts of the research range since 1983 [26]. The research range provided plots of native grass and grasslands encroached by redcedar under varied stand conditions and densities (Fig 1). The most heavily encroached area had an estimated woody canopy cover of 75% based on a plot survey done in 2010. Redcedar trees grow in three different canopy types–open stand (OS), dense stand (DS) and closed stand (CS) influenced by development stage and initial tree density. To incorporate trees growing in different canopy types, we selected a total of 5 sites—two sites for OS, one site for DS and two sites for CS (Fig 1A). Within each, five trees were selected based on their diameter at breast height (DBH) to span the range of tree sizes representative of the study area.

### Meteorological information

Meteorological information was collected by two met-stations, one located in a grassland area and one located in an opening within a redcedar encroached area. Precipitation was measured with a TB3 siphoning tipping bucket rain gage with 0.254 mm per tip (Hydrological Services America, Lake Worth, FL, USA). Average air temperature and relative humidity were measured at 2m using a Vaisala Temperature/RH Probe (HMP50, Campbell Scientific Inc, Logan, UT, USA) with a RM Young 6-plate radiation shield. Other measurements included wind speed and direction (RM Young Company Wind Sentry Set, Traverse City, MI, USA) and solar radiation (Apogee SP-110, Apogee Instruments, Inc., Logan, UT, USA) at 3-m height. The reference evapotranspiration (ET_0_) was calculated based on the standardized reference evapotranspiration equation (ASCE, 2002). From February through April, ET_0_ was calculated based on short grass. From May onward, ET_0_ was calculated based on tallgrass.

### Throughfall, stemflow, canopy storage capacity, and stemflow funneling ratio

Rainfall, throughfall and stemflow were measured for 41 rainfall events that occurred from February 2011 through the remainder of that calendar year. A rainfall event was defined as a period of measurable rainfall separated by at least four hours with no rain. This is similar to the approach used by Crouse and Corbett [27] for their grass interception study.

Throughfall for grassland vegetation was quantified using V-shape metal tray interceptors (8 trays total) (Fig 1). Tray interceptors were fabricated from aluminum roof edging with sides 3.4 cm × 3.9 cm. The metal was cut and bent to form a partial closure with an extension to which 1.27 cm ID Tygon tubing was connected to gravity route the throughfall to 2.2 liter collection vessels set in the ground. The open surface of the interceptor was covered with metal screen to prevent liter from plugging up the tubing and the tray was installed almost flush with the ground surface. Eight collar interceptors with a latex coating over the ground surface and sealing around the vegetation in order to catch stemflow along with throughfall were also installed but the data are not presented here due to problems with the latex sealing. The finished opening of the tray measured 36.5 cm × 5.0 cm (Fig 1C). The grassland site was clipped to a uniform 5 cm height in February 2011 to mimic grazing or spring burning.

Throughfall and stemflow were measured for redcedar trees in the heavily encroached areas of the study site. Throughfall for each redcedar tree was quantified using a network of 8 standard plastic rain gauges randomly distributed under the tree canopy within the canopy drip line. A total of 200 rain gauges were deployed under 25 redcedars representing different growth characteristics (Table 1). The collecting area for each rain can was 83.3 cm^2^. Throughfall depth was read directly from the rain cans and manually recorded.

**Table 1:** Characteristics of the 25 redcedar trees (*J*. *virginiana*) used in this study to represent open, dense and closed stands.

Stand	DBH (cm)	Canopy area (m^2^)
	6	9
	12	17
Open-1	22	31
	28	53
	35	49
	5	7
	8	9
Open-2	14	26
	23	27
	43	75
	6	4
	11	5
Dense	16	12
	22	14
	23	18
	6	3
	12	9
Closed-1	17	17
	25	21
	46	48
	6	3
	11	10
Closed-2	20	17
	25	30
	33	41

For stemflow, a metal collar was nailed to the trunk under the lowest branch, sealed to the tree trunk using polyurethane foam and silicone, and connected by 1.27 cm ID Tygon tubing to two sequentially connected plastic containers each with 144 liter capacity (Fig 1E and 1F). Stemflow volumes less than 2 liters were quantified manually using a graduated cylinder. For larger stemflow volumes, the water depth in the pre-calibrated collection vessel was measured with a ruler measured to the mm. In order to compare rainfall, throughfall, and stemflow depths, the collected stemflow volume was converted to stemflow depth by dividing volume by canopy area.

Canopy storage capacity (*S*) is the amount of water that can accumulate within the canopy of a tree during a rainfall event. Canopy storage capacity was calculated using the Leyton minimum method relating rainfall and throughfall [28–29].

The stemflow funneling ratio (*F*) was calculated for individual rainfall and annual rainfall based on Herwitz [30]:

$$F=\frac{V}{BA\times P}$$ (1)

where V is stemflow volume, BA is basal area, and P is the depth equivalent of event rainfall.

### Data analysis and statistics

For each rainfall event, throughfall depth for each tree was calculated as a mean value collected from 8 rain gauges. The individual tree, not the individual rain gauge, was the experimental unit. The experimental unit for grassland was the individual tray interceptor. To calculate the throughfall, stemflow or interception ratios used in the figures and statistics, throughfall and stemflow for individual trees or grass plots were summed for the time period of interest (month or annual) at the experimental unit level and divided by the sum of the rainfall depth during the same period.

Statistical analysis was performed using Proc Mixed in SAS statistical software (SAS 9.3, SAS Institute, Cary, NC). Analysis of covariance (ANCOVA) was used to test the effects of tree diameter and canopy type on annual throughfall, stemflow, and canopy interception ratios of redcedar trees. The PDIFF option was used to compare pairs of LS means for different canopy types when slopes were not different among canopy types. The non-linear relationships between stemflow funneling ratios and redcedar basal area and event rainfall amount were selected based on R^2^ in Sigmaplot 11 (Systat Software Inc., San Jose, CA, USA). Statistical analyses involving funneling ratio were conducted using ANCOVA on the log transformed values.

## Results

### Climate conditions

**Fig 2:**
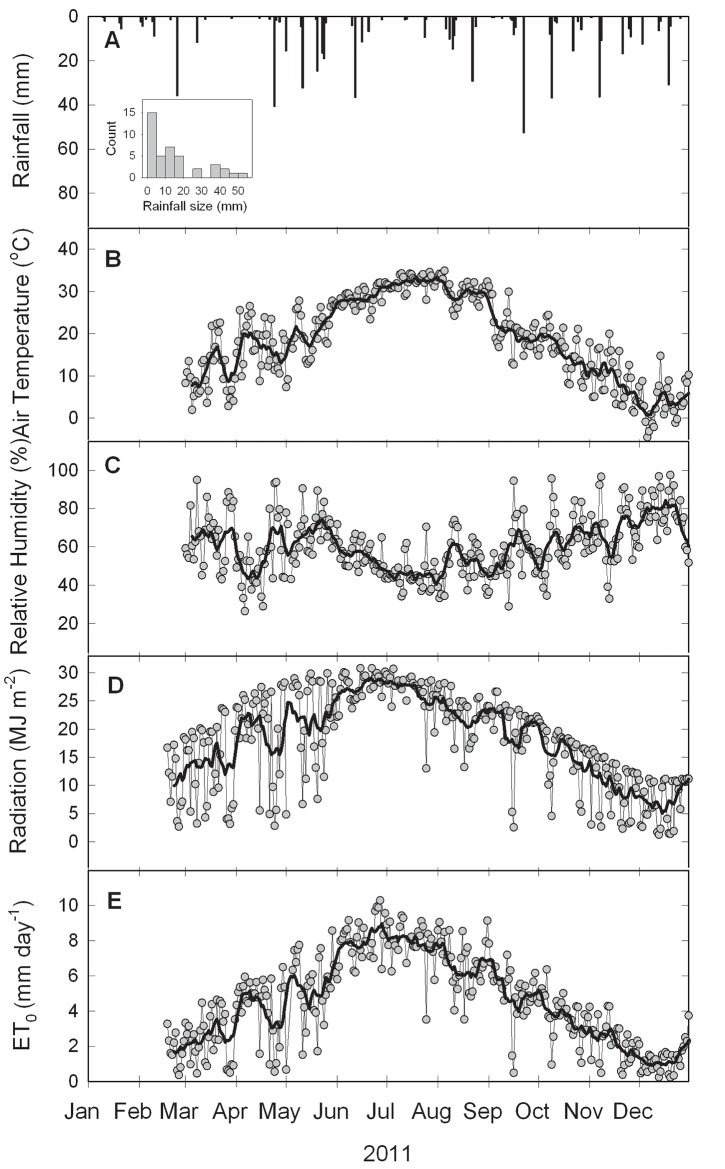
Total daily rainfall (A), average daily air temperature (B), average daily relative humidity (C), total daily radiation (D) and total daily reference ET (E) for the experimental period in 2011. The bold lines are 10-day moving averages. Inset in panel A is the histogram of frequency distribution of rainfall amounts for 41 rainfall events.

2011 was a hot and dry year (Fig 2). At our study site, late June through August had daily average air temperatures greater than 30°C and relative humidity usually below 55%. Daily ET was greater than 4 mm for most of the year. Of the 677 mm of precipitation the site received, the majority occurred as rainfall during the spring and the fall.

### Overall throughfall, stemflow, interception and rainfall

We were able to quantify throughfall, stemflow, interception and rainfall for 41 events with rainfall totaling 588 mm. Rainfall events used in the study ranged from 0.5 to 52.6 mm; precipitation from snow and ice events was not included. Fifteen of the rainfall events were smaller than 5 mm. For the 25 redcedars, average annual throughfall was 57.3% of the event rainfall with a range from 38 to 76%. The average annual stemflow for those same trees was 6.4% with a range of 1.1 to 21%. Therefore the average annual interception for redcedars was 36.3%. For the grasses we were only able to obtain throughfall values from the tray interceptors because the 8 collar interceptors with a latex coating over the ground surface and sealing around the vegetation failed to collect either throughfall or stemflow. For the year, using the data from the tray interceptors, throughfall for grass averaged 56% with a range of 30 to 80%. The average interception loss for grass using our tray interceptors was 44%.

**Fig 3:**
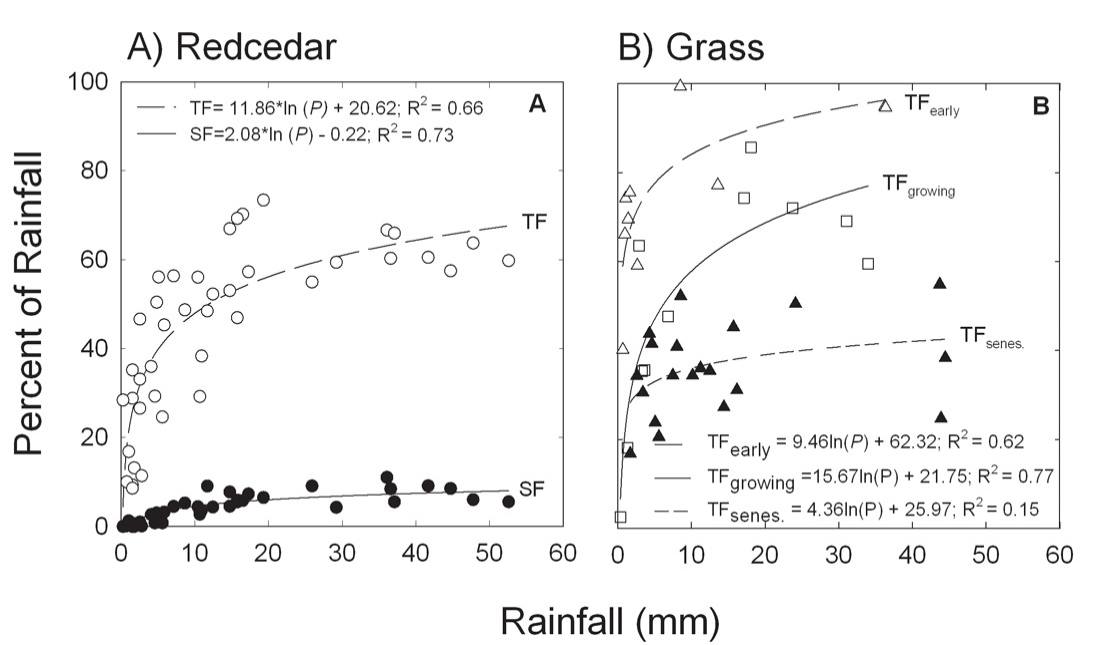
Non-linear models describing average throughfall (TF) ratios and stemflow (SF) ratios for redcedar trees (A) and TF for tallgrass prairie vegetation at different growing stages (B) under the range of event rainfall sizes.

The percent of rainfall partitioning into throughfall and stemflow increased logarithmically with increasing event amount for redcedars (Fig 3A). Rainfall amount explained 60% and 73% of the variation of measured throughfall and stemflow, respectively, for redcedars (Fig 3A). Similarly, the percent of rainfall becoming throughfall in prairie increased logarithmically with increasing rainfall amount as well (Fig 3B). Event rainfall amount accounted for 62% and 77% variation of measured throughfall for early stage and growing stage of grassland, but event rainfall size explained little variation of throughfall for the senescent stage (Fig 3B) or for the entire period (R^2^ = 0.048).

### Canopy storage capacity (S)

**Fig 4:**
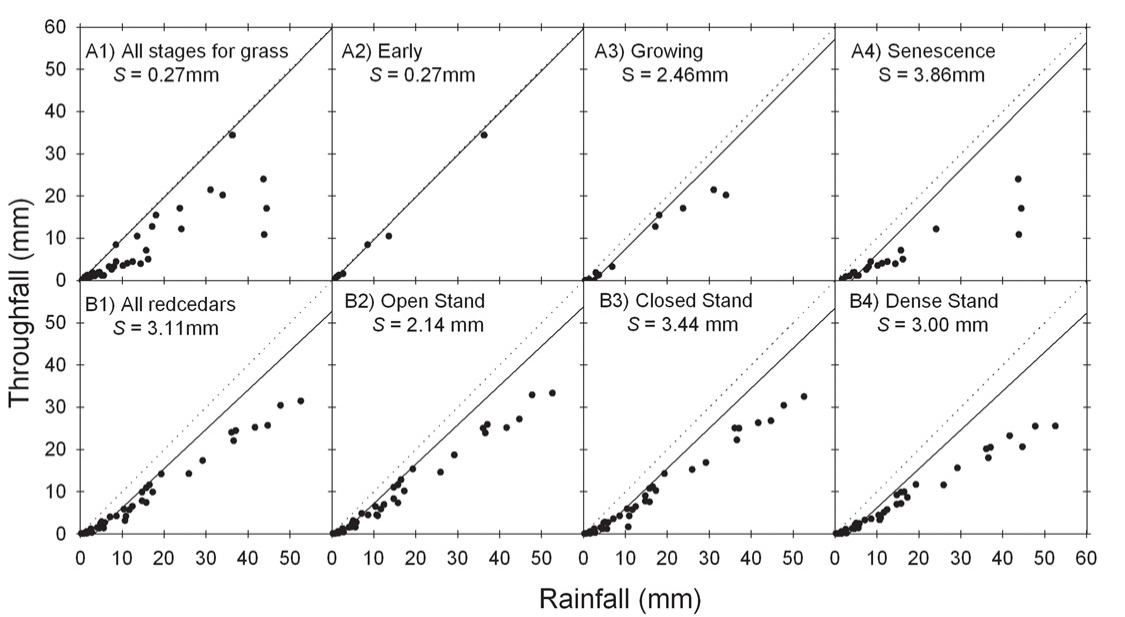
Canopy storage capacities (*S*) of prairie grasses at different growing stages (A) and of redcedar trees with different canopy structure (B). The dotted lines are the 1:1 line and the solid lines are the minimum storage envelope line.

*S* values for grasses increased with grass growth stage from *S* as small as 0.27 mm in the early growing stage to *S* as high as 3.86 mm at senescence (Fig 4A). Averaged over different DBH tree sizes, *S* values of redcedars ranged from 2.14 mm for open stands to 3.00 mm for dense stands and 3.44 mm for closed stands trees (Fig 4B).

### Stemflow funneling ratios (F) for redcedars

**Fig 5:**
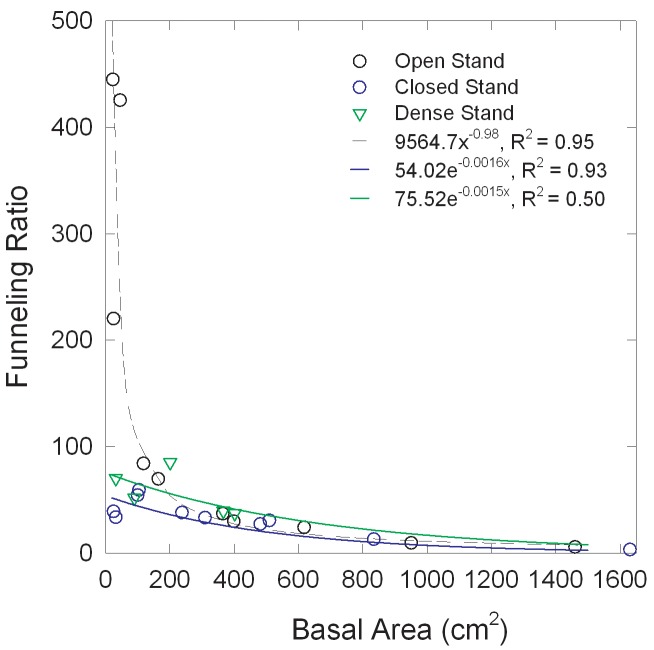
Relationship between annual stemflow funneling ratios and redcedar basal area for the different canopy types.

The calculated annual *F* for redcedars decreased with increasing basal area (Fig 5) irrespective of canopy types. However, this relationship differed between open, closed or dense stand trees (F = 3.44; df = 2.19; p = 0.05) with the log transformed slope of open stand trees steeper (-0.00285 ± 0.00034 SE) than closed stand trees (-0.00164 ± 0.00033 SE) and dense stand trees (-0.00154 ± 0.00147 SE). As a result, F was significantly greater for the open stand trees when basal area was low, i.e., open > closed at basal areas between 0 and 400 cm^2^ and open > dense at basal area near 0 cm^2^ (Fig 5).

**Fig 6:**
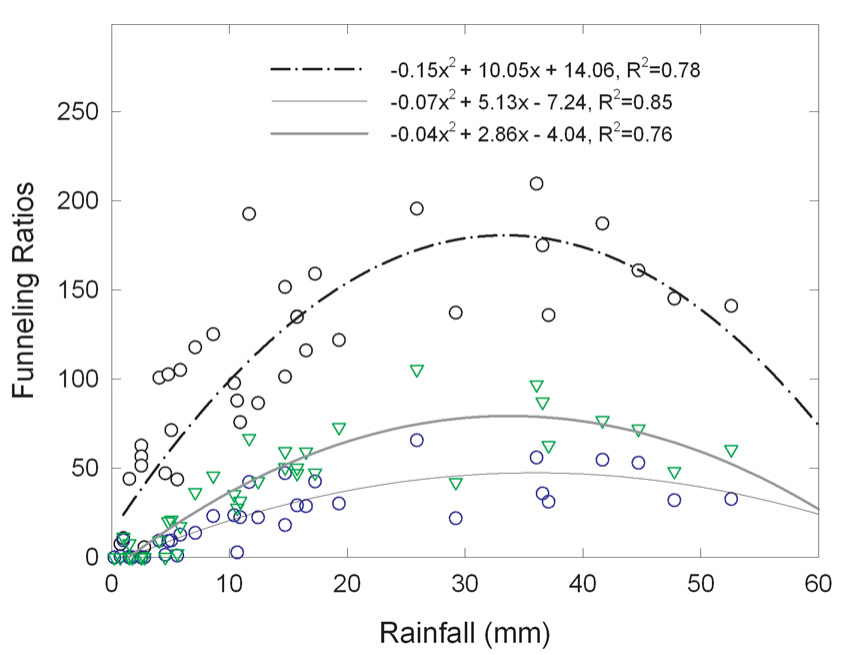
Relationships between event stemflow funneling ratios and rainfall depth for the different canopy types.

For all canopy types the funneling ratio initially increased with increasing rainfall amounts and reached peak *F* values at about 35 mm. The funneling ratio then began to decrease as rainfall amounts increased from 35 mm to 53 mm, our highest rainfall event. This trend followed a second order polynomial relationship with R^2^ values from 0.76 to 0.85 (Fig 6). Event rainfall amount explained much of the variation in funneling ratios for the three canopy types. For the full range of event rainfall amounts the open stand redcedars had the highest average funneling ratios, across all tree sizes, followed by dense, and then closed stand redcedars. Based on the linear relationship of stemflow to rainfall amount, the calculated threshold of rainfall amount to produce stemflow for open, dense and closed stands was 2.3 mm, 2.7 mm and 3.0 mm, respectively.

### Redcedar structural metrics and rainfall redistribution

**Fig 7:**
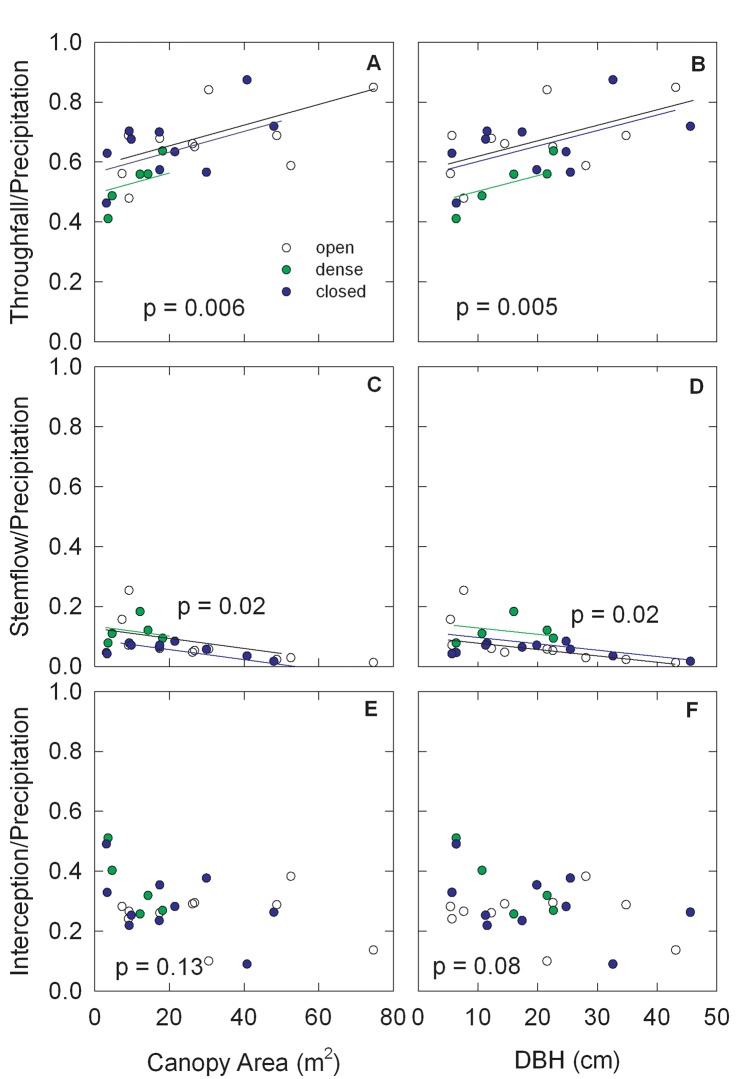
Effects of redcedar canopy area and DBH on the fraction of annual precipitation measured as throughfall and stemflow and interception for the different canopy types.

On an annual basis, throughfall ratio increased with canopy area (F = 9.55; df = 1,21; p = 0.006) and DBH (F = 10.01; df = 1,21; p = 0.005) (Fig 7A and 7B). When calculated on a canopy area basis, the mean throughfall of dense stand trees (0.571 ± 0.043 SE) was lower than closed stand trees (0.661 ± 0.029 SE) (p = 0.09) while the open stand trees were intermediate (0.641 ± 0.030 SE) (Fig 7A). When calculated at a common DBH, the mean throughfall of dense stand trees (0.549 ± 0.041 SE) was lower than the closed (0.648 ± 0.029 SE) (p = 0.06) or open (0.665 ± 0.029 SE) (p = 0.03) stand trees (Fig 7B), i.e., the relationship shifted downward for dense stand trees.

The stemflow ratios decreased with increasing canopy area (F = 9.58; df = 1,21; p = 0.006) and DBH (F = 6.41; df = 1,21; p = 0.02) (Fig 7C and 7D). When expressed on a canopy area basis, the mean stemflow of the closed stand trees (0.053 ± 0.014 SE) was lower than the open (0.089 ± 0.015 SE) (p = 0.09) and dense stands (0.098 ± 0.021 SE) (p = 0.08) trees (Fig 7C). On a DBH basis, closed stand trees had lower mean stemflow (0.059 ± 0.015 SE) than dense stand trees (0.110 ± 0.021 SE) (p = 0.06) while the open stand trees were intermediate (0.078 ± 0.015 SE) (Fig 7D).

The canopy interception ratio was not significantly related to canopy area (F = 2.43, df = 1,21; p = 0.13) (Fig 7E), but decreased with DBH (F = 3.40; df = 1,21; p = 0.08) (Fig 7F). There were no significant differences for canopy interception ratios among the canopy types. Means of canopy interception ratio expressed on a DBH basis were 0.293 ± 0.029 SE, 0.341 ± 0.042 SE and 0.256 ± 0.029 SE for the closed, dense, and open grown stands respectively. Means of canopy interception ratio expressed on a canopy area basis were 0.285 ± 0.030 SE, 0.331 ± 0.044 SE, and 0.269 ± 0.031 SE for the closed, dense, and open grown stands respectively.

### Seasonal variation and accumulated change of canopy interception

**Fig 8:**
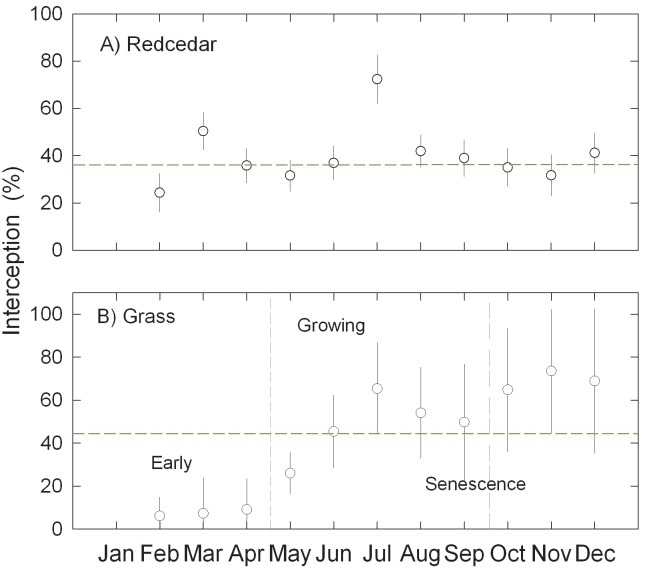
Temporal relationship of rainfall interception ratios for redcedars (A) (Error bars are standard errors, n = 5) and grassland (B) (Error bars are standard errors, n = 8).

The monthly rainfall interception ratios for redcedar trees varied with no apparent pattern (Fig 8). The highest monthly interception ratio was in July and lowest in February (Fig 8A). The monthly rainfall interception ratios for tallgrass prairie increased with the grass canopy development and senescence (Fig 8B). Less than 10% of event rainfall was lost to grass canopy between March and April. During the growing season from May to September, 20–60% of event rainfall was lost to canopy and over 60% of event rainfall was lost to canopy when grass senescence started at the late stage.

**Fig 9:**
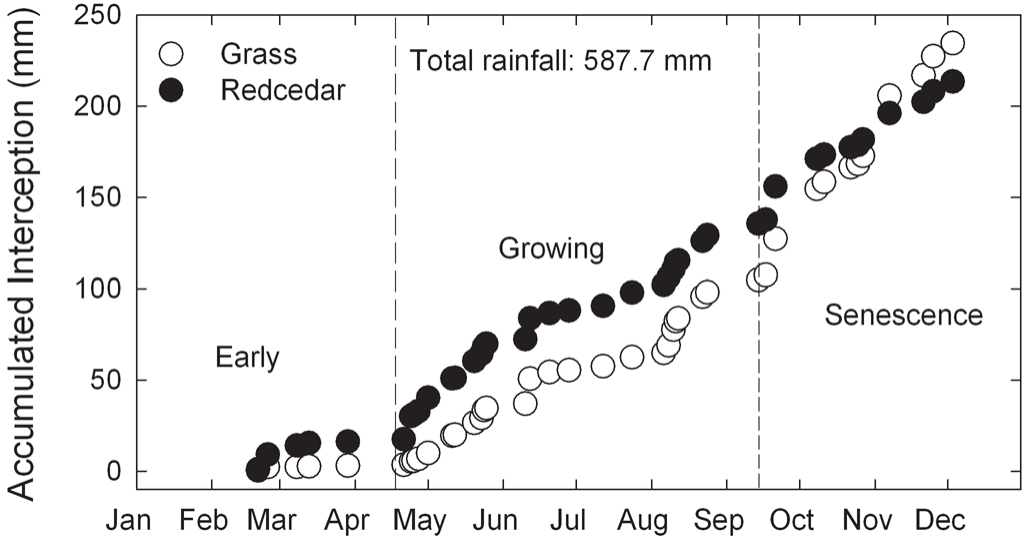
Comparison of accumulated interception loss to tallgrass prairie and redcedars.

The accumulated amount of rainfall loss to redcedar canopy was consistently higher than tallgrass prairie until grass senescence (Fig 9). By late November and early December, the accumulated amount of rainfall loss to tallgrass prairie approached and even surpassed that of redcedar (Fig 9). On annual basis, there was no significant difference in interception ratios between redcedar canopy (36.3 ± 4.3% SE) and tallgrass prairie (43.9.0 ± 5.8% SE) (F = 3.13; df = 1,31; p = 0.08).

## Discussion

Our study provides some of the first insights to understand the interception dynamics of tallgrass prairie and encroaching eastern redcedar as well as the temporal instability of canopy rainfall interception for tallgrass prairie. Specifically, our rainfall interception measurements of contrasting vegetation canopy structure and phenology under the same climatic regime yielded similar canopy interception ratios, supporting convergence in interception ratio for different canopy structures under the same precipitation regime. Below, we discuss the importance of climatic factors on canopy interception and how our redcedar canopy interception ratios fit in this broader context. Additionally we acknowledge the importance of variation in canopy funneling ratios as a function of stand density, and why inclusion of phenology and growth stage improves the quantification of canopy interception for tallgrass prairie. Finally, we will discuss the ecohydrological implication of our results.

### Climate control on canopy interception

Our study period (2011) occurred in the midst of a severe drought year for the south-central Great Plains. For the state of Oklahoma, the June through August period was the hottest since 1934 and the 3rd driest since 1936. The average rainfall was 291 mm lower, the relative humidity was 7% lower, the average air temperature was 1.11°C higher, the daily average of total solar radiation was 0.7 MJ m^-2^ higher, and the annual reference evapotranspiration (*ET*_*0*_) was 406.4 mm higher compared to the recent 15-year (1999–2013) average. The high temperature and high *ET*_*0*_ in 2011 likely produced higher *E*_*s*_ [31] and therefore higher canopy interception than normal. Likewise, the frequency of small rainfall events (Fig 2A, inset) resulted in high canopy interception due to the nonlinear relationship between interception loss and rainfall amount (Fig 3). Therefore the annual canopy interception ratios for both redcedar and tallgrass in our study may serve as an upper bound climate wise for potential water loss, rather than a mean value for each vegetation types. This result suggests increased interception loss in vegetated ecosystems for the projected warmer and drier climate in future.

It is acknowledged that precipitation regime largely dictates the interception ratio [15, 32]. For redcedar in this study, rainfall amount explained 60–70% of the variation in throughfall and stemflow ratios. This is consistent with the relationship reported for *J*. *ashei* [15] and *J*. *flaccida* [12], suggesting the relative importance of rainfall regime. *Juniperus* is a widely distributed genus through the arid and semiarid mountain west to the subhumid transition zone in the Great Plains in the USA. Even with similar canopy cover, the annual percentage of rainfall lost to juniper canopy interception can differ substantially under different climate and rainfall regimes. While rainfall amounts also accounted for much of the variation in throughfall for grass during the early and rapid growth phases, event rainfall size explained a relatively small part of the variations of throughfall during the late or senescent phase in this study. How to calculate rainfall interception after senescence or whether to include that time period will largely affect the annual interception value. This, to certain degree, explains the wide range of values reported for tallgrass prairie interception [19–20].

### Overall throughfall, stemflow, and interception

Our overall value for *J*. *virginiana* interception in this study, 36.3%, was consistent with the 40% of precipitation lost to *J*. *ashei* in Texas that was reported by Owens *et al*. [15] and slightly lower than the 52% loss that Duesterhaus [16] measured for *J*. *virginiana* in Kansas. Our average annual stemflow value, with all canopy types included, was also similar to Owens *et al*. [16] value of 5%. Our overall average grass interception value of 44% for the year falls within the wide range of values that others have reported for prairie grasses [19–20].

### Canopy storage capacity (S) and canopy characteristics

We did not find any reported water storage capacity values for juniper species in the literature for comparison. Our estimated *S* values of 2.14 to 3.44 mm for redcedar trees are in the upper range of *S* reported for *Pinus* and *Picea spp* [33], smaller compared to *S* value reported for *Pseudotsuga menziesii* [29], and substantially higher than *S* values reported for broadleaf trees [34]. *S* for our tallgrass plot was 2.46 mm during the period of full canopy development (May to October), consistent to 2.3 mm reported for a fully developed stand of big bluegrass (*Andropogon furcatus* Muhl.) [35], but substantially higher than the *S* value of 1.1 to 1.8 mm documented for mixed grass prairie [35–36]. Our *S* values at initial growing phase (0.27 mm) and senescent phase (3.86 mm) were substantially different from the period of full canopy development. However, our *S* value during senescence phase had high uncertainty. No attempt was made to remove any plant debris that might have accumulated on the surface of the tray interceptor. Therefore, our measured water storage capacity for grasses also included water loss to litter.

Several processes could explain the high water storage capacity values that we report here for both redcedar trees and prairie grasses. The Leyton method [28] assumes negligible *E*_*t*_ of the intercepted rainfall from the interception surface. In addition, foliar absorption was reported for juniper species [37] under water stress. These two processes augment “loss” of rainfall to leaves. Considering the overall hot and dry conditions for our study period (Fig 2), existence of both processes could result in a higher *S*. Some direct methods for obtaining canopy storage capacity have been reported in the literature [33, 38–39] and future studies may consider comparing *S* values by these various methods.

### Canopy funneling ratio (*F*) and tree growth characteristics

Season-long average funneling ratios for deciduous tree species typically range from 7 to 26 [40] although *F* values up to 240 have been reported for Mediterranean shrubs [41]. For the redcedars in this study, *F* values ranged mostly from 2 to 100. The three smallest open stand trees had higher *F* values of 200–450 (Fig 5). The funneling efficiency decreased with increasing basal area, similar to results reported by Murakami [42]. This could be partially correlated with the rapid decrease of the canopy area to basal area ratios with increasing tree diameter. The canopy to basal area ratios for our smallest open stand redcedar trees (DBH < 10 cm) ranged from about 2000 to 3600, but the ratios for large trees (DBH > 30 cm) dropped substantially, ranging from about 300 to 800. The funneling ratio may also decline with basal area due to the thick and rough, shredding bark, which develops on the larger redcedar trees. We did not quantify the bark water storage capacity in this study and an increase of water absorption by bark could also contribute to reduced stemflow and therefore reduced *F* values for larger diameter trees [43]. In our study the high *F* values of over 400 for the small, open stands redcedar trees are largely the result of the very large canopy spread relative to their basal area. These trees extended branches further than those of the small closed or dense stand redcedar trees. We speculate that high *F* values also result from the redcedar trees’ branching structure, where branches tend to angle upward, making them very effective in capturing and funneling rainfall towards the stem. For redcedars encroaching upon grasslands, the high stemflow and *F* values lead to localized concentration of water and nutrients around the redcedar tree trunks in the herbaceous dominated ecosystem allowing the redcedars to become established.

The relationship between *F* and event rainfall amount followed a second order polynomial relationship, which is consistent with findings of Carlyle-Moses and Price [40] and Li *et al*. [44] for other species. This pattern was explained as a result of threshold type of response [40], with increasing rainfall input, a greater proportion of a tree becomes saturated and thus the area contributing to stemflow increases until a threshold rainfall input (35 mm for redcedar in this study) is reached that saturates all tree areas capable of producing stemflow. Once this threshold rainfall is exceeded, F value begins to decrease. Other explanations for such pattern are that 1) intense rain events increase the probability of branch drip by overloading preferential flow paths on tree trunks and forcing stemflow to become throughfall [45–46] or 2) a larger proportion of intercepted precipitation was “splashed” from the canopy surfaces, thus, reducing the amount of water available for stemflow production [47].

### Phenology, growth stage, canopy architecture and accumulated canopy interception loss

Redcedar trees are evergreen with relatively small seasonal changes in leaf turnover and canopy architecture compared to grasses. As a result, the canopy architecture and interception surface of redcedar trees can be viewed as unchanged over the course of a year. It is interesting to find out in our study that the different structural metrics associated with open, closed and dense stands mostly affect the net rainfall partitioning between throughfall and stemflow, not the net interception itself. Further work to understand the ecohydrological and biochemical implications of such mechanism is needed. The high variability of *S* values (Fig 4) in tallgrass prairie reflect the high instability of grassland canopy characteristics associated with different developmental phases through the year. Interestingly, our results showed that event rainfall amount explained over 77% of the variation of observed throughfall for tallgrass prairie during the growing season, which was higher than 73% for recedar. This improved predication could be partially explained by the spatial uniformity of grass canopy and relatively consistent climatic condition under which throughfall was collected. The challenge remains for quantifying throughfall for the late phase during which time the grasses senesce. Grass senescence fundamentally alters the architecture and texture of grass canopy. Even though there may be minimal change in total biomass, gradual conversion from vertical to more horizontal structure probably increases the interception surface and may also better buffer wind turbulence, effectively reducing throughfall. In this study, we did not remove dead vegetative materials covering the throughfall interceptors to avoid disturbance of vegetation standing structure. As a result, throughfall reported at the late stage also includes rainfall loss to the litter accumulated in the same year and represents the net rainfall input into mineral soil.

The accumulated rainfall loss to canopy interception by grass was substantially lower than redcedar until the middle of September when grasses started to senesce. How to handle rainfall loss to senesced grass will largely affect the outcome of the comparison between redcedar and prairie interception loss. Two caveats are needed to interpret the annual rainfall interception results from this study. Comparison of water loss to canopy interception is usually brought up in the context of land management. Grassland excluded from fire and grazing, as in this study, does not represent the prevailing land management for tallgrass prairie. The majority of the working tallgrass prairie in the south-central Great Plains, USA is grazed and exposed to regular fire, both removing a substantial portion of biomass (live and dead) from the land surface and reducing the effective interception surface. As a result, water loss to canopy interception from a working grassland should be much lower than what we reported here. In addition, the litter layer depth and dynamic differ among redcedar and grasses. Field observations indicated a consistent litter layer of 2–4 cm under redcedar canopy. This litter layer could add another approximately 5% rainfall interception to the total rainfall interception based on a study of *J*. *ashei* [16]. Grassland litter decomposes much faster and our *in situ* observations suggest that the litter mass is negligible before senescence. Our grass throughfall data captured the litter interception for the senescence period. Finally, our attempt to quantify tallgrass stemflow using latex to seal the soil surface in a PVC collar was not successful and we do not have values for stemflow generation by native prairie grasses. Assuming grass stemflow is negligible, we would conclude that encroachment of redcedar into working rangeland will result in a substantial increase in water loss to canopy interception, especially for the early spring period when the grass canopy is not fully developed. For most of the south-central Great Plains, USA, runoff concentrates in the spring and early summer [14], therefore an increase of rainfall loss to redcedar canopy and litter could result in a reduction in soil water and runoff to streams.

### Ecohydrological implication of our results

Soil water content is both the cause and consequence of vegetation dynamics in water-limited ecosystems [48]. The amount of rainfall that bypasses the canopy layer as throughfall to replenish rooting zone moisture plays a critical role in vegetation growth [36, 48–49]. In turn, vegetation growth and, therefore, expansion of canopy reduces throughfall by increasing interception loss [11–12]. The high canopy interception surface associated with redcedar reduces the total net rainfall and soil water available for growth, but changes in canopy structure from grass to tree results in a localized concentration of water and nutrients, which provides a positive feedback to facilitate woody plant encroachment.

## Conclusions

Redcedar is a species with relatively high canopy storage capacity, and consequently has a high rainfall interception ratio. The growth characteristics of these trees, such as canopy type and trunk diameter, affect the partitioning of net rainfall into throughfall and stemflow, but growth characteristics do not significantly affect the total interception ratio; fractional canopy cover alone can be used as a scalar to estimate interception loss by redcedar at the landscape scale. The percent of rainfall that is intercepted is largely determined by event rainfall amount.

The interception by native grass prairie was also strongly influenced by rainfall amounts but varied through the year based on the stage of growth. Therefore the seasonal change of canopy structure in grassland is critically important in estimating rainfall interception. Without external management, the increasing structural complexity associated with redcedar encroachment into tallgrass prairie changes the rainfall redistribution and partitioning pattern at both the temporal and spatial scales, but does not change the overall canopy interception ratios compared with unburned and ungrazed tallgrass prairie. This finding supports the idea of convergence in interception ratio for different canopy structures under the same precipitation regime.

Resource conservation is an important ecosystem function and water is the most critical resource in semiarid and subhumid ecosystems. Without external management, the relatively high and similar total percent loss of rainfall to canopy from both herbaceous and woody plants, in the subhumid ecosystem is an interesting finding from this study. As a result, the temporal stability of high rainfall interception from redcedar throughout the year might be more critical in altering streamflow response than its total interception loss. The relatively low interception ratios at the early phase and growing phase by grass coincide with the important streamflow generation period in the system. Therefore this temporal evolution in canopy development associated with grassland is a key component for the effectiveness of rainfall in bypassing the vegetative layer to replenish rooting zone moisture and enhancing vegetation growth. Further study should focus on how the temporal stability of high rainfall interception of the evergreen life form of *J*. *virginiana* will alter water budget in the south-central Great Plains.
